# Impaired Skeletal Muscle Regeneration Induced by Macrophage Depletion Could Be Partly Ameliorated by MGF Injection

**DOI:** 10.3389/fphys.2019.00601

**Published:** 2019-05-17

**Authors:** Xiaoguang Liu, Zhigang Zeng, Linlin Zhao, Peijie Chen, Weihua Xiao

**Affiliations:** ^1^School of Kinesiology, Shanghai University of Sport, Shanghai, China; ^2^College of Physical Education, Jinggangshan University, Jiangxi, China

**Keywords:** MGF, muscle regeneration, inflammatory cytokines, oxidative stress factors, chemokines, matrix metalloproteinases

## Abstract

Skeletal muscle injury is one of the most common injuries in sports medicine. Our previous study found that macrophage depletion impairs muscle regeneration and that mechano growth factor (MGF) may play an important role in this process. However, whether injection of MGF protects against impaired muscle regeneration after macrophage depletion has not been explored. Therefore, we generated a muscle contusion and macrophage depletion mouse model and injected MGF into the damaged muscle. Comprehensive morphological and genetic analyses were performed on the injured skeletal muscle after macrophage depletion and MGF injection. The results showed that injection of MGF did not exert a protective effect on muscle fiber regeneration; however, it did decrease fibrosis in the contused skeletal muscle after macrophage depletion. Moreover, MGF injection decreased the expression of muscle inflammatory cytokines (TNF-α, IFN-γ, IL-1β, and TGF-β), chemokines (CCL2, CCL5, and CXCR4), oxidative stress factors (gp91phox) and matrix metalloproteinases (MMP-1, MMP-2, MMP-9, MMP-10, and MMP-14). These results suggest that the impairment of skeletal muscle regeneration induced by macrophage depletion could be partly ameliorated by MGF injection and that inflammatory cytokines, oxidative stress factors, chemokines, and MMP may be involved in this process.

## Introduction

Skeletal muscle accounts for almost half of the body weight in humans. As a power-generating tissue, it is one of the most important organs in mammals. Skeletal muscle injuries occur very often in humans and are inevitable. Skeletal muscle regeneration following an injury is a complicated process that involves many cells and cytokines (Tidball, 2011).

Inflammation, especially macrophage inflammation, plays a crucial role in skeletal muscle regeneration (Kharraz et al., 2013). Experiments have revealed that skeletal muscle injury induces extensive macrophage infiltration at the injury site (Novak et al., 2014). There are two different phenotypes of macrophages, M1 and M2. Classically activated macrophages, typically designated M1 macrophages, are characterized by the expression of proinflammatory cytokines (IL-1β and TNF-α), high expression of the CD68 surface marker and absence of the CD163 marker (Pillon et al., 2013). These M1 macrophages infiltrate injury sites following muscle damage and are involved in the phagocytosis of muscle debris (Novak and Koh, 2013). Then, the M1 macrophages are replaced by “alternatively activated” macrophages (M2 phenotype), which are characterized by high expression of CD163 and anti-inflammatory cytokines (such as IL-10) and contribute to muscle regeneration (Pillon et al., 2013; Shono et al., 2013).

Studies have shown that macrophages not only act as regulators of inflammation but also as secretory cells that produce many cytokines, such as IL-1β, IL-10, IGF-1, HGF, uPA and mechano growth factor (MGF) (Novak et al., 2011; Pillon et al., 2013; Saclier et al., 2013; Sawano et al., 2014; Sun et al., 2018). Our previous study indicated that MGF expression in the macrophages of overload training rats increased significantly compared to expression levels in the control animals (Xiao et al., 2013). A recent study found that macrophages are the main contributors to the upregulation of MGF in injured skeletal muscle (Sun et al., 2018). Furthermore, our previous study found that macrophage depletion significantly decreased the expression of MGF and the regeneration of impaired skeletal muscle (Liu et al., 2016). However, whether injection of MGF protects against impaired muscle regeneration after macrophage depletion has not been explored. We hypothesize that the injection of MGF at least partially protects against impaired skeletal muscle regeneration after macrophage depletion.

**FIGURE 1:**
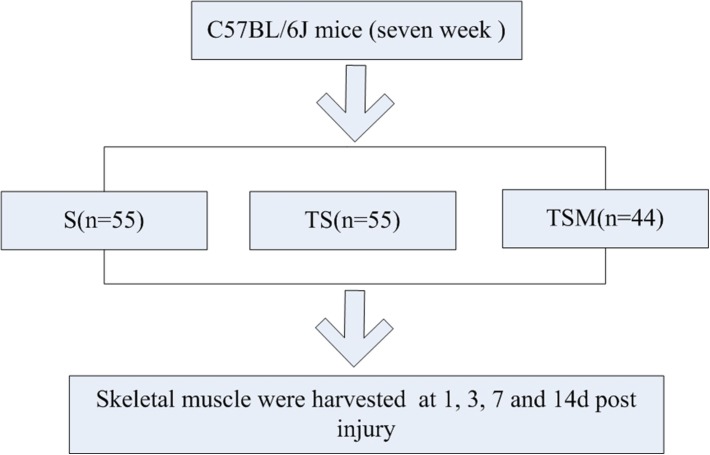
Experimental schedule. S group, skeletal muscle contusion group; TS group, skeletal muscle contusion and macrophage depletion group; TSM group, muscle contusion, macrophage depletion, and MGF treated group.

To explore this concept, we generated a muscle contusion and macrophage depletion mouse model and injected MGF into the damaged muscle of the mice. The histomorphology, fibrosis and expression of inflammatory cytokines, chemokines, oxidative stress factors, and matrix metalloproteinases (MMPs) were tested in the injured skeletal muscle (Figure 1).

## Materials and Methods

### Animals

One hundred fifty-four C57BL/6J mice [7 weeks old, Shanghai Laboratory Animal Center (SLAC), Co., Ltd.] were used in this study. After acclimatization to the local environment for 1 week, the mice were divided into three groups: skeletal muscle contusion mice (S group), skeletal muscle contusion and macrophage depletion mice (TS group), muscle contusion, macrophage depletion and MGF-treated mice (TSM group). The animals were housed at a constant temperature of 25°C with free access to pellet food and water. The study and all protocols were approved by the Ethics Review Committee for Animal Experimentation of Shanghai University of Sport.

### Skeletal Muscle Contusion Model

The mouse gastrocnemius muscle (GM) contusion model was generated according to the methods used in our previous study (Liu et al., 2016; Xiao et al., 2016b). Briefly, animals were anesthetized via intraperitoneal injection of 35 mg/kg pentobarbital sodium. The knee was extended, and the ankle was plantar-flexed at 90° after the mice were anesthetized. A 16.8 g (diameter: 15.9 mm) stainless steel ball was dropped from a height of 125 cm through a plastic tube with an interior diameter of 16 mm onto an impactor.

### Macrophage Depletion

To generate a macrophage depletion model, clodronate-containing liposomes^1^ were used as previously described (Popovich et al., 1999; Tyner et al., 2005; Summan et al., 2006). Briefly, 2 mg of clodronate-containing liposomes (CL) or control liposomes was introduced by intrapulmonary injection 3 days before the contusion injury. A total of 0.5 mg of CL or control liposomes was then injected at 0, 3, 6, 9, and 12 days after the muscle was contused. This macrophage depletion protocol was effective and has been used by many researchers (Popovich et al., 1999; Tyner et al., 2005; Summan et al., 2006; Liu et al., 2016; Xiao et al., 2016a).

### MGF Treatment

A total of 5 μg/20 μl MGF peptide (GL Biochem, Ltd., Shanghai; Lot No. P140627-25-LR066649) was injected into each GM immediately and at 1 and 2 days after muscle injury. The control mice were injected with PBS-BSA buffer (Mills et al., 2007; Philippou et al., 2008).

### Hematoxylin and Eosin (H&E) Staining

After the mice were sacrificed, the GMs were collected and placed in 4% paraformaldehyde before routine paraffin embedding. For the morphometric analysis, 5-μm cross sections of GMs were generated with a microtome (Leica-EG 1160, Germany) and were deparaffinized and stained with H&E. Using the 40× lens objective of a light microscope (Labphot-2, Nikon), image was captured for each muscle section.

### Masson’s Trichrome Staining

Masson’s trichrome staining was used to measure the fibrotic tissue area in the injured GMs. After the staining, ImageJ software (ImageJ 1.44, Bethesda, MD, United States) was used to estimate the fibrotic area of the injured skeletal muscle. The sum of the fibrotic area was quantified as previously described (Xiao et al., 2016a).

### Immunofluorescence Staining

Identification of gp91phox in injured muscle was performed by immunofluorescence staining of the GMs. Slides of the GM sections were incubated with a bovine serum albumin (BSA) blocking buffer for 1.5 h and with the gp91phox primary antibody (Abcam, 1:1000) overnight at 4°C. The slides were washed three times in PBS, for 5 min per wash. The sections were incubated with a goat anti-rabbit IgG (Abcam, 1:800) secondary antibody for 60 min, washed three times in PBS, and incubated with 4′,6-diamidino-2-phenylindole (Beyotime Biotech, Co., Ltd.) for 5 min. Using a 20× lens objective (LSM700, Zeiss), an image was captured for each muscle section. Immunofluorescence staining intensity was semiquantitatively analyzed by ImageJ 1.44 image analysis software (National Institutes of Health, Bethesda, MD, United States).

### RNA Extraction, cDNA Synthesis, and Real-Time Polymerase Chain Reaction (PCR)

Total RNA was isolated from the GMs using the guanidinium isothiocyanate-CsCl method as previously described (Xiao et al., 2013). Total RNA (2 μg) was reverse-transcribed into cDNA (Revertaid^TM^ First Strand cDNA Synthesis Kit, Thermo Scientific). Q-PCR was performed on a StepOnePlus PCR-Cycler (Life Technologies) using the SYBR Green/ROX qPCR Master Mix (Vazyme Biotech, Co., Ltd.) with the following components: 1 μl of cDNA, 7.8 μl of nuclease-free water and 300 nM of each primer. The activation step was performed at 95°C for 10 min, which was followed by 40 cycles of denaturation at 95°C for 15 s and annealing/extension at 60°C for 1 min. The relative mRNA expression was determined using the 2^ΔΔCT^ method (Livak and Schmittgen, 2001). The primer sequences are listed in Table 1.

**Table 1:** Primers used for qRT-PCR.

Target gene	Forward primer sequences	Reverse primer sequences
TNF-α	5′-CTTCTGTCTACTGAACTTCGGG-3′	5′-CACTTGGTGGTTTGCTACGAC-3′
INF-γ	5′-GCTTTGCAGCTCTTCCTCAT-3′	5′-GTC ACC ATCCTTTTGCCAGT-3′
IL-1β	5′-TGACGTTCCCATTAGACAACTG-3′	5′-CCGTCTTTCATTACACAGGACA-3′
TGF-β	5′-TGCGCTTGCAGAGATTAAAA-3′	5′-CGTCAAAAGACAGCCACTCA-3′
Col1a1	5′-GAGCGGAGAGTACTGGATCG-3′	5′-GCTTCTTTTCCTTGGGGTTC-3′
Col3a1	5′-GTCCACGAGGTGACAAAGGT-3′	5′-GATGCCCACTTGTTCCATCT-3′
MMP-1	5′-AGTTGACAGGCTCCGAGAAA-3′	5′-CACATCAGGCACTCCACATC-3′
MMP-2	5′-ACCCTGGGAGAAGGACAAGT-3′	5′-ATCACTGCGACCAGTGTCTG-3′
MMP-9	5′-CGTCGTGATCCCCACTTACT-3′	5′-AACACACAGGGTTTGCCTTC-3′
MMP-10	5′-GAGTGTGGATTCTGCCATTGA-3′	5′-TCTCCGTGTTCTCCAACTGC-3′
MMP-14	5′-CCTGGCTCATGCCTACTTCC-3′	5′-GCACAGCCACCAAGAAGATG-3′
CCL2	5′-GCTCAGCCAGATGCAGTTAAC-3′	5′-CTCTCTCTTGAGCTTGGTGAC-3′
CCR2	5′-GAAAAGCCAACTCCTTCATCAG-3′	5′-TCTAAGCACACCACTTCCTCTG-3′
CCL5	5′-CATATGGCTCGGACACCA-3′	5′-ACACACTTGGCGGTTCCT-3′
MyoD	5′-GAGCGCATCTCCACAGACAG-3′	5′-AAATCGCATTGGGGTTTGAG-3′
myogenin	5′-CCAGTACATTGAGCGCCTAC-3′	5′-ACCGAACTCCAGTGCATTGC-3′
F4/80	5′-AACATGCAACCTGCCACAAAC-3′	5′-ACAGGATTCGTCCAGGC-3′
CXCR4	5′-CAAGGCCCTCAAGACGACAG-3′	5′-CCCCCAAAAGGATGAAGGAG-3′
gp91phox	5′-CCAGTGAAGATGTGTTCAGCT-3′	5′-GCACAGCCAGTAGAAGTAGAT-3′
GAPDH	5′-ACTCCACTCACGGCAAATTC-3′	5′-TCTCCATGGTGGTGAAGACA-3′


### Statistical Analysis

The data were analyzed using SPSS 20.0 software for Windows (IBM, United States). The number, diameter and area of regenerating myofibers or fibrotic regions were compared using independent samples *t*-tests. A factorial analysis of the multi-way ANOVA with the one-way ANOVA was used to analyze the main effects of the S, TS, and TSM groups. *Post hoc* Scheffe tests were performed when significance was detected by the one-way and multi-way ANOVA tests. All values are expressed as the mean ± SEM, and statistical significance was set at *p* < 0.05.

## Results

### Effect of Clodronate-Containing Liposomes on Macrophage Marker in Injured Skeletal Muscle

**FIGURE 2:**
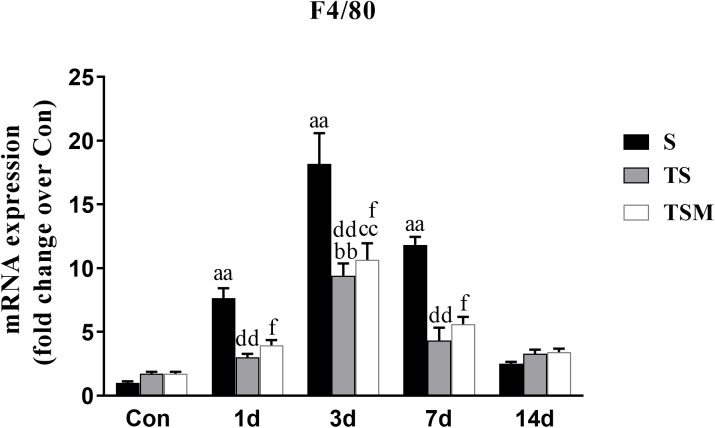
Effects of clodronate-containing liposomes one marker of macrophage in injured skeletal muscle. S, muscle contusion group; TS, muscle contusion and macrophage depleted group; TSM, muscle contusion, macrophage depletion and MGF treated group. Data are means ± SEM, *n* = 8; ^a^Significant difference from Scon, *p* < 0.05; ^aa^*p* < 0.01. ^b^Significant difference from TScon, *p* < 0.05; ^bb^*p* < 0.01. ^c^Significant difference from TSMcon, *p* < 0.05; ^cc^*p* < 0.01; ^d^Significant difference between group TS and group S, *p* < 0.05; ^dd^*p* < 0.01; ^e^Significant difference between group TS and group TSM, *p* < 0.05; ^ee^*p* < 0.01; ^f^Significant difference between group TSM and group S, *p* < 0.05; ^ff^*p* < 0.01.

RT-PCR results indicated that clodronate-containing liposomes significantly decreased the expression of the F4/80 macrophage marker at 1, 3, and 7 days (*p* < 0.01) post-skeletal muscle injury. In addition, the injection of MGF did not influence expression of the macrophage marker after treatment of injured skeletal muscle with clodronate-containing liposomes (*p* > 0.05) (Figure 2).

### MGF Injection Did Not Exert a Protective Effect on Muscle Fiber Regeneration After Macrophage Depletion

**FIGURE 3:**
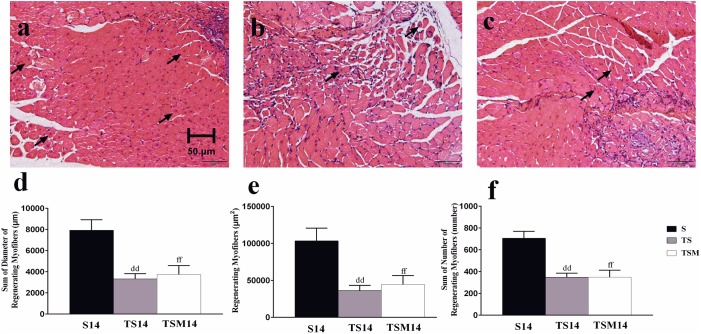
Effects of MGF injection on muscle regeneration after macrophage depletion at 14 days post-injury. **(a)** Muscle contusion group (14 days post-injury); **(b)** muscle contusion and macrophage depleted group (14 days post-injury); **(c)** muscle contusion, macrophage depleted, and MGF treated group (14 days post-injury); **(d)** quantification of the diameter of regenerating myofibers in GMs; **(e)** quantification of the area of regenerating myofibers in GMs; **(f)** quantification of the number of regenerating myofibers in GMs; Data are means ± SEM, *n* = 4. ^dd^Significant difference from S14, *p* < 0.01; ^ff^Significant difference from S14, *p* < 0.01. Scale bars = 50 μm. regenerating myofibers.

H&E staining showed that macrophage depletion significantly decreased the sums of the diameters (7927 ± 991.20 vs. 3318.76 ± 503.19 μm, *p* < 0.01), numbers (706 ± 63.64 vs. 348 ± 37.15, *p* < 0.01) and areas (103,461.79 ± 17,145.27 vs. 36,055.28 ± 71,57.94 μm^2^, *p* < 0.01) of the regenerating myofibers at 14 days post-injury. However, unexpectedly, MGF injection had no effect on the sums of the diameters, numbers and areas of the regenerating myofibers (*p* > 0.05) (Figure 3d–f).

### MGF Injection Decreased the Fibrosis of Contused Skeletal Muscle After Macrophage Depletion

**FIGURE 4:**
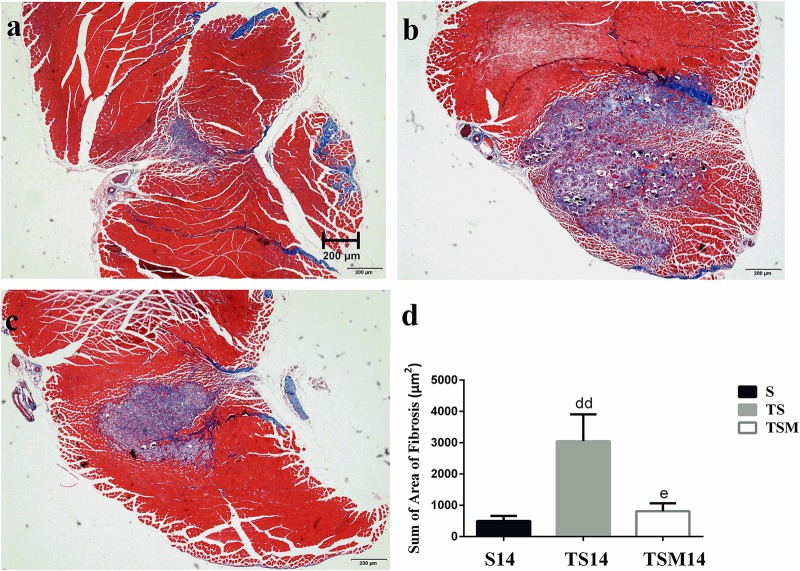
Effects of MGF injection on muscle fibrosis after macrophage depletion at 14 days post-injury. **(a)** Muscle contusion group (14 days post-injury); **(b)** muscle contusion and macrophage depleted group (14 days post-injury); **(c)** muscle contusion, macrophage depletion, and MGF treated group (14 days post-injury); **(d)** quantification of the fibrosis area in GMs (14 days post-injury); Data are means ± SEM, *n* = 3. ^dd^Significant difference from S14, *p* < 0.01; ^e^Significant difference from TS14, *p* < 0.05. Scale bars = 200 μm. Fibrosis tissues were shown in *blue* and muscle are in *red*.

Masson’s trichrome staining was performed to evaluate whether MGF injection improved GM fibrosis after macrophage depletion in injured skeletal muscle. Masson’s trichrome staining showed that fibrosis increased significantly in the GMs in the TS group compared with the fibrosis in the S group at 14 days post-injury (496.34 ± 162.06 vs. 3047.86 ± 857.20 μm^2^, *p* < 0.01). However, interestingly, the fibrotic area decreased significantly after MGF injection compared with the fibrotic area of the TS group at 14 days post-injury (3047.86 ± 857.20 vs. 809.44 ± 254.23 μm^2^, *p* < 0.05) (Figure 4a–d).

**FIGURE 5:**
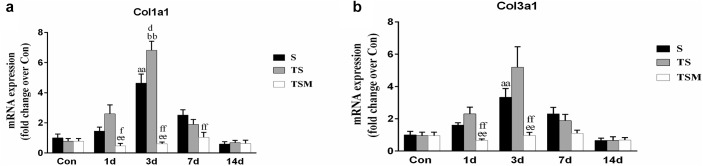
Effects of MGF treatment on the expression of collagen. **(a)** The expression of Col1a1; **(b)** The expression of Col3a1. S, muscle contusion group; TS, muscle contusion and macrophage depleted group; TSM, muscle contusion, macrophage depletion and MGF treated group. Data are means ± SEM, *n* = 8; ^a^Significant difference from Scon, *p* < 0.05; ^aa^*p* < 0.01. ^b^Significant difference from TScon, *p* < 0.05; ^bb^*p* < 0.01. ^c^Significant difference from TSMcon, *p* < 0.05; ^cc^*p* < 0.01; ^d^Significant difference between group TS and group S, *p* < 0.05; ^dd^*p* < 0.01; ^e^Significant difference between group TS and group TSM, *p* < 0.05; ^ee^*p* < 0.01; ^f^Significant difference between group TSM and group S, *p* < 0.05; ^ff^*p* < 0.01.

In addition, we tested the mRNA expression of collagen I and III in injured skeletal muscle. The results revealed that MGF injection significantly decreased the expression of collagen I mRNA levels at 1 day (*p* < 0.01) and 3 days (*p* < 0.01) after muscle injury compared with the TS group (Figure 5a). The expression of collagen III mRNA decreased in the MGF-treated mice at 1 (*p* < 0.01) and 3 days (*p* < 0.01) post-injury compared with the collagen III mRNA levels of the TS group (Figure 5b).

### MGF Injection Did Not Influence the Functional Status of Satellite Cells in Injured Skeletal Muscle After Macrophage Depletion

**FIGURE 6:**
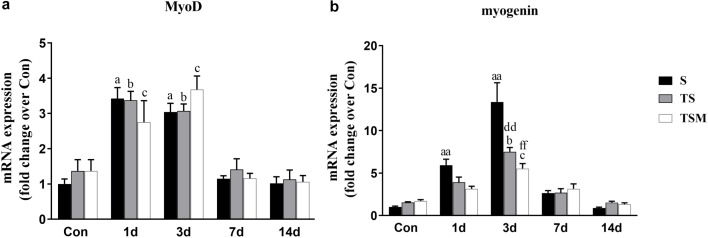
Effects of MGF treatment on the expression of MyoD and myogenin. **(a)** The expression of MyoD; **(b)** The expression of myogenin. S, muscle contusion group; TS, muscle contusion and macrophage depleted group; TSM, muscle contusion, macrophage depletion and MGF treated group. Data are means ± SEM, *n* = 8; ^a^Significant difference from Scon, *p* < 0.05; ^aa^*p* < 0.01. ^b^Significant difference from TScon, *p* < 0.05; ^bb^*p* < 0.01. ^c^Significant difference from TSMcon, *p* < 0.05; ^cc^*p* < 0.01; ^d^Significant difference between group TS and group S, *p* < 0.05; ^dd^*p* < 0.01; ^e^Significant difference between group TS and group TSM, *p* < 0.05; ^ee^*p* < 0.01; ^f^Significant difference between group TSM and group S, *p* < 0.05; ^ff^*p* < 0.01.

Myogenic differentiation antigen (MyoD) is an important factor for muscle regeneration and is considered a marker of proliferation in satellite cells, and myogenin is a marker of differentiation in satellite cells (Cornelison and Wold, 1997). In this study, we found that macrophage depletion did not influence the expression of MyoD (Figure 6a) but did significantly decrease the expression of myogenin at 3 days (*p* < 0.01) post-muscle injury (Figure 6b). However, injection of MGF did not influence the expression of MyoD and myogenin in the injured skeletal muscle after macrophage depletion (Figure 6a,b).

### MGF Injection Decreased Expression of Inflammatory Cytokines in Injured Skeletal Muscle After Macrophage Depletion

**FIGURE 7:**
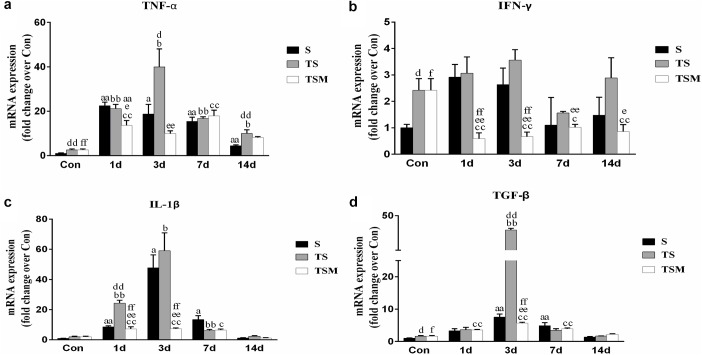
Effects of MGF treatment on the expression of inflammatory cytokines in injured skeletal muscle after macrophage depletion. **(a)** The expression of TNF-α; **(b)** The expression of IFN-γ; **(c)** The expression of IL-1β; **(d)** The expression of TGF-β. S, muscle contusion group; TS, muscle contusion and macrophage depleted group; TSM, muscle contusion, macrophage depletion, and MGF treated group. Data are means ± SEM, *n* = 8; ^a^Significant difference from Scon, *p* < 0.05; ^aa^*p* < 0.01. ^b^Significant difference from TScon, *p* < 0.05; ^bb^*p* < 0.01. ^c^Significant difference from TSMcon, *p* < 0.05; ^cc^*p* < 0.01; ^d^Significant difference between group TS and group S, *p* < 0.05; ^dd^*p* < 0.01; ^e^Significant difference between group TS and group TSM, *p* < 0.05; ^ee^*p* < 0.01; ^f^Significant difference between group TSM and group S, *p* < 0.05; ^ff^*p* < 0.01.

RT-PCR demonstrated that levels of pro-inflammatory cytokines (TNF-α, IL-1β, and TGF-β) were significantly higher in the TS group than in the S group 3 days after the skeletal muscle injury. In contrast, the injection of MGF resulted in significantly decreased TNF-α, IFN-γ, IL-1β, and TGF-β levels in the TSM group post-injury compared with the TS group (Figure 7a–c).

### MGF Injection Regulated Chemokine Expression in Injured Skeletal Muscle After Macrophage Depletion

**FIGURE 8:**
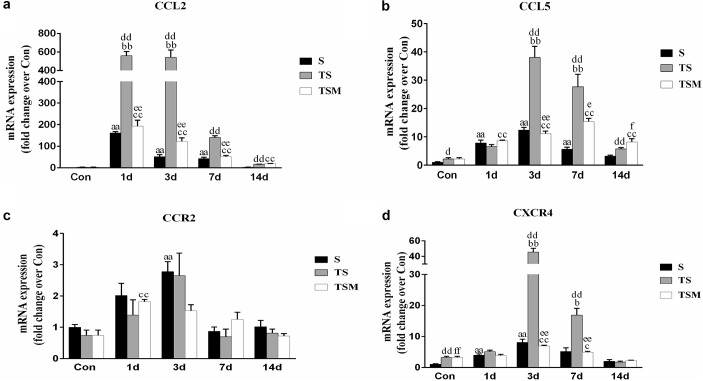
Effects of MGF injection on the expression of chemokines in injured skeletal muscle after macrophage depletion. **(a)** The expression of CCL2; **(b)** The expression of CCL5; **(c)** The expression of CCR2; **(d)** The expression of CXCR4. S, muscle contusion group; TS, muscle contusion and macrophage depleted group; TSM, muscle contusion, macrophage depletion and MGF treated group. Data are means ± SEM, *n* = 8; ^a^Significant difference from Scon, *p* < 0.05; ^aa^*p* < 0.01. ^b^Significant difference from TScon, *p* < 0.05; ^bb^*p* < 0.01. ^c^Significant difference from TSMcon, *p* < 0.05; ^cc^*p* < 0.01; ^d^Significant difference between group TS and group S, *p* < 0.05; ^dd^*p* < 0.01; ^e^Significant difference between group TS and group TSM, *p* < 0.05; ^ee^*p* < 0.01; ^f^Significant difference between group TSM and group S, *p* < 0.05; ^ff^*p* < 0.01.

Chemokines are important cytokines in skeletal muscle regeneration and fibrosis (Grefte et al., 2010; Brzoska et al., 2012). Compared with the S group, the group of mice with macrophage depleted muscle showed higher levels of CCL2, CCL5, and CXCR4 at 3 and 7 days post-injury (Figure 8a,b,d). Compared with the TS group, the MGF-treated group showed significantly decreased expression levels of CCL2, CCL5, and CXCR4 at 3 and 7 days post-injury. However, there was no significant change in the expression of CCR2 mRNA in the TSM group compared with the TS group post-injury (*p* > 0.05) (Figure 8c).

### MGF Injection Decreased the Expression of gp91phox in Injured Skeletal Muscle After Macrophage Depletion

**FIGURE 9:**
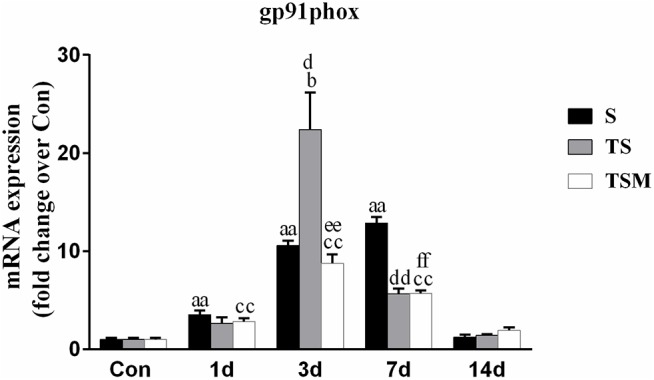
Effects of MGF injection on the expression of gp91phox in injured skeletal muscle after macrophage depletion. S, muscle contusion group; TS, muscle contusion and macrophage depleted group; TSM, muscle contusion, macrophage depletion and MGF treated group. Data are means ± SEM, *n* = 8; ^a^Significant difference from Scon, *p* < 0.05; ^aa^*p* < 0.01. ^b^Significant difference from TScon, *p* < 0.05; ^bb^*p* < 0.01. ^c^Significant difference from TSMcon, *p* < 0.05; ^cc^*p* < 0.01; ^d^Significant difference between group TS and group S, *p* < 0.05; ^dd^*p* < 0.01; ^e^Significant difference between group TS and group TSM, *p* < 0.05; ^ee^*p* < 0.01; ^f^Significant difference between group TSM and group S, *p* < 0.05; ^ff^*p* < 0.01.

**FIGURE 10:**
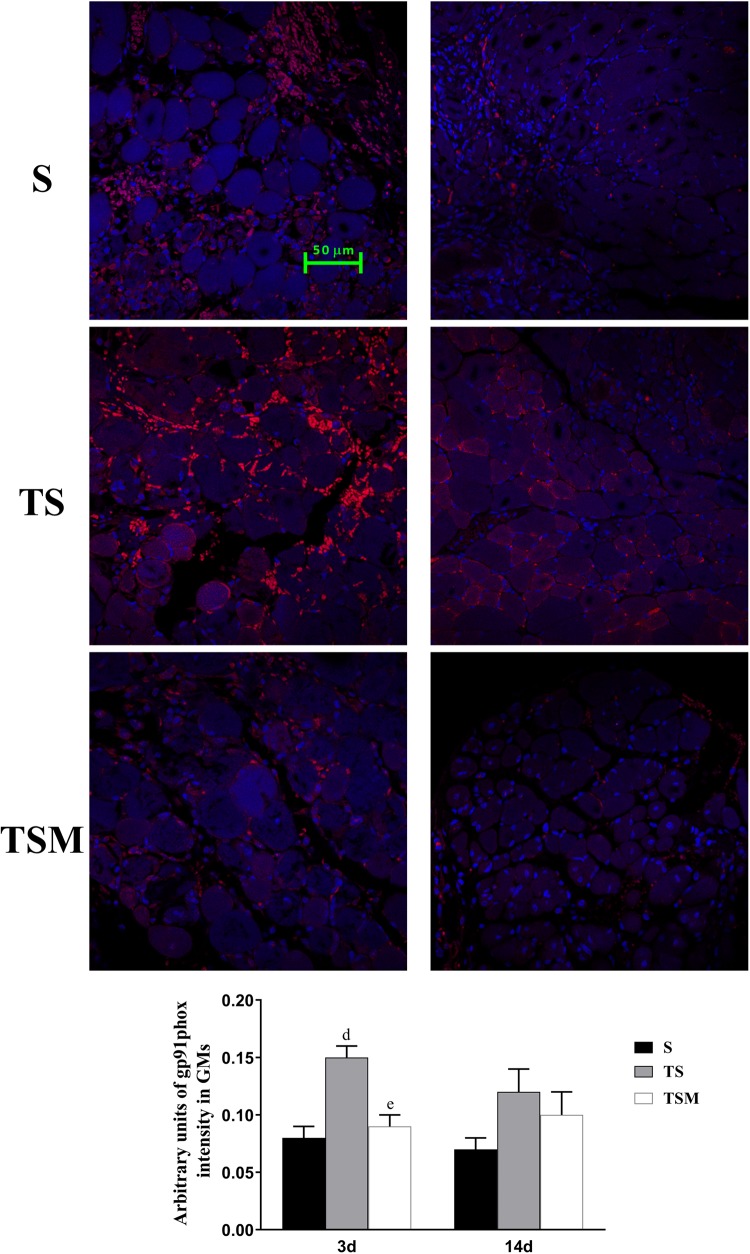
Effects of MGF injection on the expression of gp91phox in injured skeletal muscle after macrophage depletion. S, muscle contusion group; TS, muscle contusion and macrophage depleted group; TSM, muscle contusion, macrophage depletion and MGF treated group. Data are means ± SEM, *n* = 8; ^a^Significant difference from Scon, *p* < 0.05; ^aa^*p* < 0.01. ^b^Significant difference from TScon, *p* < 0.05; ^bb^*p* < 0.01. ^c^Significant difference from TSMcon, *p* < 0.05; ^cc^*p* < 0.01; ^d^Significant difference between group TS and group S, *p* < 0.05; ^dd^*p* < 0.01; ^e^Significant difference between group TS and group TSM, *p* < 0.05; ^ee^*p* < 0.01; ^f^Significant difference between group TSM and group S, *p* < 0.05; ^ff^*p* < 0.01.

Oxidative stress contributes to fibrotic scar formation after skeletal muscle injury (Ghaly and Marsh, 2010). In this study, we tested whether MGF injection improves oxidative stress in injured skeletal muscle after macrophage depletion. The RT-PCR results showed that MGF injection significantly decreased the expression of gp91phox, a key subunit of NADPH oxidases (Chan et al., 2009), in the TSM group compared with the TS group at 3 and 14 days post-injury (*p* < 0.05) (Figure 9). In addition, the immunofluorescence staining showed similar results (Figure 10).

### MGF Injection Decreased the Expression of Matrix Metalloproteinases in Injured Skeletal Muscle After Macrophage Depletion

**FIGURE 11:**
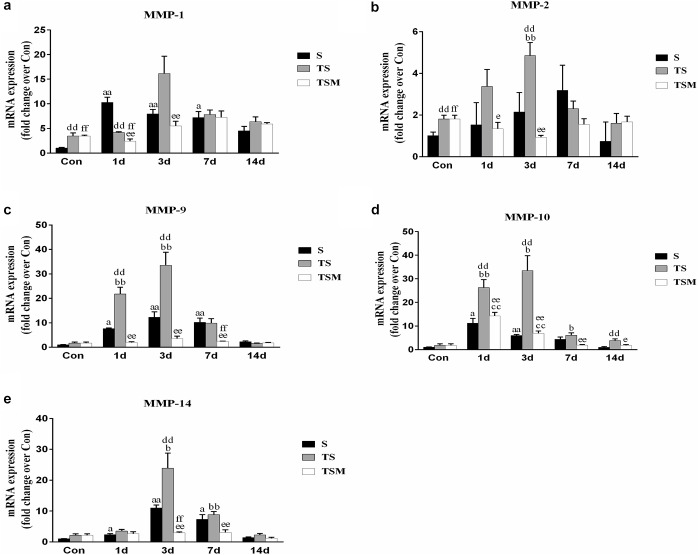
Effects of MGF treatment on the expression of MMP. **(a)** The expression of MMP-1; **(b)** The expression of MMP-2; **(c)** The expression of MMP-9; **(d)** The expression of MMP-10; **(e)** The expression of MMP-14. S, muscle contusion group; TS, muscle contusion and macrophage depleted group; TSM, muscle contusion, macrophage depletion and MGF treated group. Data are means ± SEM, *n* = 8; ^a^Significant difference from Scon, *p* < 0.05; ^aa^*p* < 0.01. ^b^Significant difference from TScon, *p* < 0.05; ^bb^*p* < 0.01. ^c^Significant difference from TSMcon, *p* < 0.05; ^cc^*p* < 0.01; ^d^Significant difference between group TS and group S, *p* < 0.05; ^dd^*p* < 0.01; ^e^Significant difference between group TS and group TSM, *p* < 0.05; ^ee^*p* < 0.01; ^f^Significant difference between group TSM and group S, *p* < 0.05; ^ff^*p* < 0.01.

Macrophage depletion caused significant increases in MMP-1, MMP-2, MMP-9, MMP-10, and MMP-14 levels in injured skeletal muscle compared with the MMPs levels in the S group after injury. Compared with the TS group, the group that received the injection of MGF exhibited significantly increased levels of skeletal muscle MMP-1, MMP-2, MMP-9, MMP-10, and MMP-14 (*p* < 0.01) at 3 days after muscle injury (Figure 11a–e).

## Discussion

To investigate the protective effect of MGF in impaired skeletal muscle regeneration after macrophage depletion and the underlying mechanisms involved, we generated a muscle contusion and macrophage depletion mouse model. In our previous study, we found that injection of clodronate-containing liposomes was an effective method to deplete the macrophage in injured skeletal muscle (Xiao et al., 2016a). Consistent with that study, we found that injection of clodronate-containing liposomes significantly decreases the expression of a macrophage marker (F4/80) (Figure 2). These results suggested that macrophages were effectively depleted. In addition, we found that MGF injection has no influence on the expression of the macrophage marker (F4/80) after clodronate-containing liposomes were injected into injured skeletal muscle.

The H&E results indicated that macrophage depletion impairs muscle regeneration and that MGF injection exerts no protective effect, as evidenced by the sum of the diameters, numbers, and areas of the regenerating muscle fibers (Figure 3). These results are consistent with those of Sun et al. (2018), who found that MGF overexpression did not notably affect muscle regeneration outcomes.

Furthermore, we tested the effects of MGF treatment on the fibrosis of injured muscle after macrophage depletion. Masson’s trichrome staining results showed that MGF injection significantly decreased the fibrotic area in the GMs in the TSM group compared with the fibrotic area in the TS group at 14 days post-injury (Figure 4). The fibrotic areas of the injured skeletal muscles exhibited markedly increased levels of collagen types I and III in the ECM of the muscle (Huebner et al., 2008; Lieber and Ward, 2013). Sha et al. (2017) indicated that exogenous MGF-E peptide reduced collagen type I/III synthesis in cultured anterior cruciate ligament fibroblasts. Consistent with the aforementioned result, the levels of collagens I and III decreased significantly in the TSM group compared with the levels in the TS group at 1 and 3 days post-injury (Figure 5a,b). This result suggests that MGF injection can decrease the fibrosis of injured muscle after macrophage depletion.

To study the mechanism mediating in these results, we examined the expression of MyoD (a marker of proliferation of satellite cells) and myogenin (a marker of differentiation of satellite cells) (Cornelison and Wold, 1997). Our results showed that injection of MGF did not influence the expression of MyoD and myogenin in the injured skeletal muscle after macrophage depletion (Figure 6a,b). These results suggest that MGF injection improves skeletal muscle fibrosis after macrophage depletion but not by regulating the functional status of satellite cells.

Furthermore, we examined the expression of inflammatory cytokines after muscle injury. Previous studies have shown that TGF-β plays a central role in skeletal muscle fibrosis (Zhu et al., 2007; Lieber and Ward, 2013). Our data showed that MGF treatment significantly decreased the expression of TGF-β mRNA in the TSM group compared with the expression in the TS group at 3 days post-injury (*p* < 0.01, Figure 7d). This result may explain why MGF injection significantly decreases muscle fibrosis after macrophage depletion. Furthermore, we analyzed the expression of IL-1β, TNF-α, and IFN-γ mRNA after muscle injury. Some inflammatory cytokines (such as IL-1β, TNF-α, and TGF-β) are significantly higher after macrophage depletion in injured skeletal muscle, which may come from other inflammatory cells (Panduro et al., 2018). Furthermore, our results showed that MGF treatment significantly decreased the levels of IL-1β, TNF-α, and IFN-γ (Figure 7a–c) in the TSM group compared with the levels in the TS group. These results were similar to the findings of another study that showed that exogenous MGF-E peptide downregulates the protein levels of IL-1β and TNF-α in fibroblast-like synoviocytes in osteoarthritis (Li et al., 2015). The present results suggest that MGF injection improves skeletal muscle fibrosis after macrophage depletion by decreasing the expression of pro-inflammatory cytokines (TNF-α, IL-1β, and TGF-β).

Chemokines are not only involved in the migration and activation of monocytes, neutrophils, macrophages and lymphocytes but also play a key role in muscle regeneration (Warren et al., 2004). In this study, we found that macrophage depletion significantly increased the expression of chemokines (CCL2, CCL5 and CXCR4) (Figure 8a,b,d). However, MGF injection significantly decreased the fibrosis of injured muscle tissue after macrophage depletion and decreased the expression of the three chemokines (CCL2, CCL5, and CXCR4). In other disease models, chemokines are involved in the fibrosis process of injured tissues, much like CCL5 contributes to liver fibrosis in non-alcoholic fatty liver disease progression (Li et al., 2017) and CXCR4 contributes to cardiac fibrosis in diabetes (Chu et al., 2015). This result suggests that the decreased levels of chemokines may be conducive to improving muscle fibrosis after MGF injection.

Because NADPH oxidase-induced reactive oxygen species (ROS) are involved in the fibrosis of injured skeletal muscle (Cabello-Verrugio et al., 2011; Kozakowska et al., 2015), we analyzed the expression of gp91phox, a key subunit and common marker of NADPH oxidase (Chan et al., 2009). The results showed that the injection of MGF significantly decreased the expression of gp91phox in the TSM group compared with the expression in the TS group at 3 days post-injury (*p* < 0.01) (Figure 9). Several studies using gene knockout mice or pharmacological agents to deplete gp91phox resulted in significantly reduced fibrosis in the kidney, liver, and heart (Chan et al., 2009). This result suggests that gp91phox may be involved in the process underlying the improved muscle fibrosis after macrophage depletion as a result of MGF treatment.

In addition, we investigated the expression of MMPs, which play important roles in skeletal muscle regeneration and fibrosis. It has been acknowledged that MMPs can electively digest the extracellular matrix (ECM) in physiological and pathological states (Chen and Li, 2009). Inappropriate ECM digestion contributes to muscle fibrosis. The mRNA levels of MMPs (MMP-1, MMP-2, MMP-9, and MMP-10) (Figure 11a–d) were significantly downregulated in the MGF-treated group after macrophage depletion in the injured muscle compared to the MMP mRNA levels in the TS group. In other muscle injury disease models (such as dystrophic muscle in mdx mice), MMPs are significantly upregulated, whereas inhibitors of MMPs alleviate the related pathology and improve skeletal muscle function (Kumar et al., 2010). This observation may be help explain why the injection of MGF improves muscle fibrosis.

## Conclusion

The results suggest that the impairment of skeletal muscle regeneration induced by macrophage depletion could be partly ameliorated by MGF injection and that inflammatory cytokines, oxidative stress factors, chemokines and matrix metalloproteinases may be involved in the process.

## Ethics Statement

The study was approved by the Ethics Review Committee for Animal Experimentation of Shanghai University of Sport, Shanghai, China (Reference No. 2014025).

## Author Contributions

WX and PC designed this study and helped to draft the manuscript. XL carried out data analysis and drafted the manuscript. XL, ZZ, and LZ performed the histological staining and carried out the real time PCR. All authors have read and approved the final version of the manuscript, and agreed with the order of presentation of the authors.

## Conflict of Interest Statement

The authors declare that the research was conducted in the absence of any commercial or financial relationships that could be construed as a potential conflict of interest.

## Footnotes

1 www.clodronateliposomes.com
